# Effectiveness of a clinical guideline to improve dental health among orthodontically treated patients: study protocol for a cluster randomized controlled trial

**DOI:** 10.1186/s13063-016-1325-1

**Published:** 2016-04-15

**Authors:** Barbara C. M. Oosterkamp, Afzal Wafae, Jan G. J. H. Schols, Wil J. M. van der Sanden, Michel Wensing

**Affiliations:** Department of Orthodontics and Craniofacial Biology, Radboud University Medical Center, PO Box 9101, 6500 HB Nijmegen, The Netherlands; College of Oral Science, Quality and Safety of Oral Health Care, Radboud University Medical Center, Nijmegen, The Netherlands; Scientific Institute for Quality of Health Care, Radboud University Nijmegen Medical Center, Nijmegen, The Netherlands

**Keywords:** Guideline, Oral health, Orthodontic treatment, Study protocol

## Abstract

**Background:**

White spot lesions (WSLs) occur as a side effect in over 25 % of patients who undergo orthodontic treatment, causing aesthetic problems and a risk of deeper enamel and dentine lesions. Dutch orthodontists show substantial variation in their application of WSL preventive measures, which include little incorporation of evidence from the literature. We recently developed an evidence-based clinical practice guideline (CPG) on this topic, which was further converted into a computerized clinical decision support system (CDSS) to facilitate its incorporation into clinical practice. The present study aimed to assess the effectiveness of this CPG-based CDSS, with regard to actually preventing WSL development during orthodontic treatment with fixed appliances compared to usual preventive measures. Our study also aimed to evaluate the effects of implementing the CPG-based CDSS into routine clinical practice using a multifaceted strategy.

**Methods/design:**

We designed a hybrid effectiveness-implementation study assessing both clinical effectiveness of the CPG and its implementation into routine practice. A total of 840 patients nested in 14 orthodontic practices will be randomly assigned as clusters to the intervention or the control arm. Patients recruited by the orthodontist in the intervention group will be treated following the CPG, while the usual preventative measures will be followed in the control arm. The primary outcome measure is the proportion of patients with newly formed or enlarged WSLs after 6–9 months of treatment with fixed appliances, and at the end of treatment, using the CPG for WSL prevention compared with usual preventive measures. An additional aim is to obtain some preliminary outcomes regarding the implementation process.

**Discussion:**

This study investigates the effectiveness of a newly developed guideline to improve oral health during orthodontic treatment, while simultaneously illuminating potential difficulties in adopting a guideline in general orthodontic practice. The innovative features of this study include the risk-based CDSS that discriminates between patients’ oral health statuses with regard to preventive measure utilization in general orthodontic practices. Most studies focusing on WSL prevention apply the preventive intervention to each patient in an experimental setting, resulting in overtreatment and a disconnect from the real-world conditions in which the intervention is to be applied. Additionally, one of the overreaching goals of this initiative is to create a gold standard for WSL prevention during orthodontic treatment, against which future studies can compare new promising preventive measures and the readiness of clinicians to change and adopt new treatments. By doing so, we want to help bridge the gap between science and orthodontic clinical practice and improve the quality of oral health care.

**Trial registration:**

This trial is registered with the Dutch Trial Registry of the Dutch Cochrane Center under number NTR5012, registration date 2 March 2015.

## Background

About 53–57 % of 12-year-old Dutch juveniles undergo orthodontic treatment representing about 100,000 patients each year. Most of these patients are treated using fixed orthodontic appliances comprising metal or ceramic attachments (orthodontic brackets) that are bonded to the outer surface of the tooth and connected with wires that exert the forces required to align individual teeth. It is difficult to clean the region surrounding the bracket, making this area prone to developing dental disease or caries. Caries result when a biofilm covers an area of tooth and metabolic events within this biofilm lead to localized chemical dissolution of the tooth surface [1]. Therefore, there exists a strong relationship between caries incidence and oral hygiene among orthodontic patients compared to patients not undergoing orthodontic treatment [2].

Additionally, over 25 % of orthodontic patients show clinically visible enamel demineralization following orthodontic treatment [3]. Such enamel demineralization is considered an early stage of dental caries and is commonly referred to as a white spot lesion (WSL) due to the white appearance caused by changes in light scattering of the decalcified porous enamel. WSLs may become noticeable around the brackets within a month of bracket placement, and the opaque color seriously compromises aesthetics after debonding of orthodontic fixed appliances [4]. Although remineralization is possible, WSLs are often permanent, presenting a lifelong aesthetic problem as well as the risk of lesion progression [3, 5].

WSLs are clearly a relevant clinical problem related to treatment with fixed orthodontic appliances, and their prevention is an important concern. Numerous preventive strategies have been developed, including the maintenance of a good oral hygiene regimen involving the use of different types of mouth rinses, toothpastes, and varnishes. However, the literature describing the effectiveness of such procedures is equivocal, and largely includes study designs with high risks of bias [6] with frequent problems relating to randomization, blinding, incomplete data, and short-term follow-up [7]. Thus, orthodontists find it difficult to incorporate the existing evidence on effective preventive measures into their routine practice, and 68 % are in favor of guideline development [8].

Our research group recently developed a clinical practice guideline (CPG) on the prevention of WSLs based on the best available evidence and following a Delphi procedure to reach consensus on issues for which sound evidence is lacking [9]. However, a CPG can only impact patient outcomes if it is properly implemented into clinical practice. To facilitate the implementation of our CPG, we converted it into a computerized clinical decision support system (CDSS). A CDSS is designed to assist clinicians and health professionals with decision-making tasks, especially when the decision to initiate treatment depends on multiple patient variables, as was true for the good practice guideline (GPG) in the present study [10]. The present study aimed to assess the effectiveness of our CPG converted into a CDSS, with regard to actually preventing WSL development during orthodontic treatment with fixed appliances compared to usual preventive measures. An additional aim is to obtain preliminary outcomes on the process of CPG implementation.

## Methods/design

### Study design

We have designed a hybrid type 1 effectiveness-implementation study to primarily focus on assessing the guideline’s clinical effectiveness, while simultaneously collecting some preliminary information regarding implementation of the guideline into routine practice. The primary aim of this approach is to examine the effects of the clinical intervention (CPG) on relevant outcomes (the proportion of patients with new or enlarged WSLs), while an additional aim is to obtain preliminary outcomes on the process of CPG implementation [11].

Practitioners will be divided into two groups using cluster randomization at the level of the orthodontic practices. Patients recruited by orthodontists in the intervention group will be treated following the CPG (see intervention section), while patients recruited by orthodontists in the control arm will be treated using standard preventive measures. The proportions of patients showing newly developed or enlarged WSLs will be compared between treatment groups after 6–9 months of treatment with fixed appliances, as well as after completion of orthodontic treatment and removal of the fixed appliances.

### Primary hypothesis

The trial will evaluate the hypothesis that the preventive measures recommended by the CPG will outperform standard preventive measures with regard to the primary outcome—which is the proportion of patients with newly developed or enlarged WSLs after 6–9 months of treatment, and at the completion of treatment with fixed orthodontic appliances, with an effect size of 0.4.

### Outcome of process evaluation

The following outcomes will be analyzed to evaluate the implementation process:

- Professionals show a positive attitude regarding use of the CPG for WSL prevention
- Professionals comply with the key recommendations for WSL prevention in the CPG
- Patients of the intervention clinics show a positive attitude regarding the applied preventive strategies.

### Randomization

Utilization of both the CPG and the usual preventive measures within the same practice would inevitably result in some control patients receiving some CPG-recommended care, which would dilute the effect of the intervention. To prevent such contamination of interventions between study groups, we will perform cluster randomization at the level of practices rather than individuals. Using a software program, practices will be assigned to either the *intervention arm* or the *control arm* by an independent statistician who is not familiar with the practices.

### Ethical approval

Our study protocol has been approved by the research ethics committee of the Radboud University Nijmegen Medical Center (Centrale Commissie Mensgebonden Onderzoek). The trial will be conducted following the principles of the declaration of Helsinki, and is registered with the Dutch trial registry of the Dutch Cochrane Center.

### Study population and recruitment

#### Orthodontic practices

A convenience sample of 14 orthodontists will be asked to participate in the present trial. All selected orthodontists are members of the Dutch Orthodontic Society, and work in private practices in different geographical areas of the Netherlands. In the Netherlands, a median-size practice has four to five chairs, and a mean of 40 to 60 patients receiving full fixed appliances per month. The present trial will include only practices with at least four chairs, in accordance with our aim of testing the CPG effectiveness and implementation in regular orthodontic practices. Orthodontists will be excluded if they are unwilling to take pre- and post-treatment intra-oral digital photographs. Informed consent will be obtained from all orthodontists working in each participating practice.

#### Patients

The participating orthodontists will recruit patients, following specific instructions designed to avoid selection bias. Patients in the intervention group will receive written information describing the preventive measures that will be applied. This information will be included as part of the written informed consent for orthodontic treatment that will be obtained during the treatment plan discussion prior to starting active treatment. In both groups, both patients and parents will provide written informed consent for the use of patient records and distribution of questionnaires.

#### Inclusion and exclusion criteria

All patients between 12–18 years of age, with fully erupted permanent dentition, and who are scheduled for treatment with full fixed appliances, will be eligible to participate in this study.

Exclusion criteria are as follows:

- Patients younger than 12 years of age, since they generally do not have fully erupted dentition, and patients older than 18 years of age, since the risk of WSL development is reduced after this age
- Patients diagnosed with bronchial asthma, due to the use of fluoride varnish (Duraphat 50 mg/mL; Pharbil Waltrop GmbH, Germany, RVG 10942)
- Patients with physical or mental problems that make them incapable of practicing proper oral hygiene
- Patients who refuse to use the prescribed preventive products
- Patients with missing incisors, canines, and/or premolars, since these are the teeth scored for outcome assessment.
- Patients with cleft lip and palate or craniofacial anomalies, since different preventive strategies are needed for these patients.

#### Sample size and power calculation

We have calculated the number of patients needed to detect a difference in the proportions of patients with newly developed or enlarged WSLs between the intervention group using the CPG and the control group using standard preventive strategies. Earlier studies show that more than 1 in 4 orthodontic patients develop at least one new WSL during the course of orthodontic treatment [3]. Assuming a power of 80 %, a significance level of 5 %, and an ICC of 0.05 to correct for clustering, we calculated that we must include a minimum of 14 orthodontic practices with 50 patients per practice to detect a medium effect size of 0.4 for reduction in newly developed WSLs. The effect size was arbitrarily chosen, as no previous data establish what would constitute a clinically relevant change. The study is powered to detect an effect of 15 % absolute reduction in prevalence of WLS, under the assumptions specified above. Assuming a drop-out rate of 20 %, we plan to recruit a total of 60 patients per participating practice, resulting in a total inclusion of 840 patients. In a regular orthodontic practice, about 40 patients receive full fixed appliances each month. To account for an overestimation of the number of patients to potentially be included in the practices, we will have a 2-month inclusion period.

### Preventive intervention

**Fig. 1:**
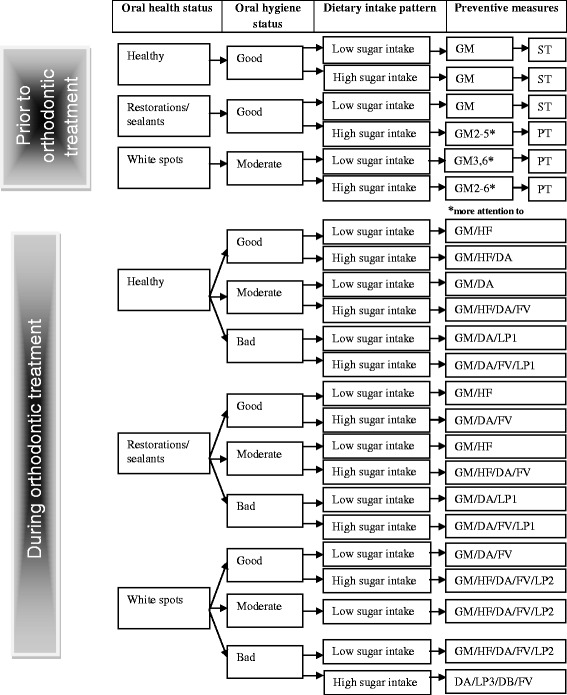
Clinical practice guideline. Abbreviations are defined in Table 1

The intervention involves the implementation of a CPG for WSL prevention prior to and during orthodontic treatment with fixed appliances (Fig. 1 and Table 1). The CPG has been converted into a CDSS that indicates the appropriateness of starting, postponing, or ending orthodontic treatment and/or of undertaking additional preventive measures during treatment. These decisions are based on the patient’s initial caries risk, as determined based on oral health status, oral hygiene status, and sugar intake. These factors will be assessed by the clinician during regular orthodontic check-up visits, which generally occur every 4–6 weeks. Based on the patient’s individual caries risk prior to orthodontic treatment, a decision will be made regarding whether to start or postpone orthodontic treatment. During orthodontic treatment, the same caries risk assessment will be used to determine whether the patient requires 1) appropriate oral hygiene instruction, 2) appropriate oral hygiene instruction and fluoride varnish application, or 3) removal of orthodontic appliances. The CPG also recommends that the orthodontist inform parents and referring dentists about the oral health situation and the possible implications for treatment.

**Table 1:** Abbreviations used in the clinical practice guideline

General preventive measures/recommendations prior to orthodontic treatment with fixed appliances	Abbreviation
1. The mouth should be free of plaque, caries, and gingivitis before starting orthodontic treatment	GM1-7
2. Explanation of the current oral health situation	
3. Advice regarding tooth-friendly nutrition and oral hygiene	
4. Oral hygiene instruction, evaluation of psychomotor skills and, when appropriate, advise brushing with powered tooth brush	
5. Topical fluoride varnish on visible/present white spots	
6. Tooth brushing with toothpaste containing fluoride (1,450 ppm) twice daily	
Start treatment when oral health status is appropriate	ST
Postpone treatment until specific requirements are met, as stated in the general measures/recommendations, including appropriate oral hygiene level and lower risk of caries due to decreased sugar intake	PT
Specific preventive measures/recommendations during orthodontic treatment with fixed appliances	
Use of a toothpaste containing fluoride (1,450 ppm) twice daily	HF
Removal of the wires, cleaning/polishing the teeth, and applying topical fluoride varnish	FV
Appropriate dietary and oral hygiene advice	DA
Letter to the (parents of the) patient including an explanation of the problem and a copy to the GDP	LP1
Letter to the (parents of the) patient describing visible carious damage to the teeth, including an explanation of the problem and a copy to the GDP	LP2
Letter to the (parents of the) patient regarding debonding (removal of all fixed appliances) with a copy to the GDP	LP3
Debonding (removal of all fixed appliances without placement of permanent retention)	DB

The CPG has been converted into a CDSS, which is to be integrated in the electronic patient files (EPFs) of the participating practices. For each patient, the program will automatically lead the clinician through the questions needed to determine the patient’s caries risk. Upon input of the answers, the program will display the recommended preventive strategy to be followed, and will store all of this information in the EPF.

The control group will not receive any intervention. Practices in the control arm of the trial will use their standard WSL preventive measures. Registration in the EPF will be performed in accordance with the medical record keeping guidelines of the Dutch Dental Society [12].

Pre- and post-treatment digital intra-oral photographs will be taken in both the intervention and the control practices. The photographic setting will be standardized in terms of lighting conditions and camera positioning to ensure validity of the WSL measurements [13].

### Implementation program

The CPG will be implemented using a multifaceted intervention strategy (See Table 2.) The implementation process will be evaluated in only a limited number of practices, since this is not the primary aim of the study. First, the CPG will be disseminated by mail to the participating orthodontists in the intervention arm, together with instructions for using the CPG. One week later, the principal investigator will make an educational outreach visit to the participating practices in the intervention arm, with the goals of further explaining the recommendations in the CPG along with their rationale, and of demonstrating the use of the CDSS integrated in the EPF. Clinicians will also be provided instructions regarding the following:

- Evaluation of oral health in terms of the presence or absence of cavities, restorations, or WSLs
- Evaluation of oral hygiene level in terms of plaque index [14], using pictures of patients with varying plaque levels
- How to appropriately explain the patient’s current oral health situation, and to give appropriate advice regarding tooth-friendly nutrition and oral hygiene
- How to give appropriate oral hygiene instruction
- How to correctly apply fluoride varnish (Duraphat 50 mg/mL for dental use) after wire removal and tooth polishing

**Table 2:** Implementation strategy

	Type of intervention
0	Dissemination of the CPG together with instructions for use
1 week	Educational outreach visits
5 weeks	Monitoring and feedback regarding use of the CPG through computerized patient records

The practices in the intervention group will also be given patient information folders containing the recommended oral hygiene and dietary instructions, together with the recommended letters that are to be sent to the parents and the general dentists in cases of poor oral hygiene. After 5 weeks, the number and types of preventive actions registered in the EPF will be extracted from the software and analyzed. This information will be used to give feedback to the participating practices regarding guideline adherence. For this purpose, a second visit by the principal investigator is planned, during which the practitioners will have the opportunity to discuss potential problems. This will provide insight into the problems and barriers encountered in the specific practice that may be hindering adequate guideline implementation.

### Outcome parameters

#### Primary outcome

The primary outcome measure is the proportion of patients treated with full fixed orthodontic appliances who develop at least one new or enlarged WSL after 6–9 months of treatment with fixed appliances, and at the completion of treatment, when using the CPG for WSL prevention as compared to usual preventive measures.

#### Outcomes for implementation process evaluation

The process of CPG adoption in routine clinical orthodontic practice will be assessed in terms of the following factors:

- Professionals’ attitudes towards utilizing the CPG for WSL prevention, measured using a questionnaire
- Professionals’ compliance with the key recommendations in the CPG for WSL prevention, measured by auditing the EPF
- Patients’ views regarding the provided preventive care, measured using a questionnaire.

### Measurements

#### Primary outcome

The number of WSLs per patient will be determined by examination of maxillary incisors and canines, mandibular canines, and maxillary and mandibular premolars by two independent observers, who will be blinded to the patient’s intervention status. Standardized pre-treatment intra-oral photographs (T0) will be compared with intra-oral photographs taken after 6–9 months of treatment (T1), and after completion of treatment with fixed orthodontic appliances (T2). If comparison of the T0 photographs to the T1 and T2 photographs reveals an identical white spot on all three images, it will be considered a developmental white spot and will not be counted as a WSL. For each patient, the total number of new and enlarged WSLs will be registered. The proportion of patients showing new or enlarged WSLs will be determined for each group.

A pilot study using pre-and post-treatment intra-oral pictures of 150 patients from three orthodontic practices (50 patients per practice) demonstrated an inter-observer agreement of 0.6, which is considered acceptable. However, this agreement varied among the practices, mainly due to the quality of the intra-oral photographs. In the retrospective pilot study, the photographic technique was not standardized in terms of lighting and camera positioning. For the presently described prospective trial, lighting and camera positioning will be standardized, which will likely lead to better agreement. An earlier *in vitro* study measured artificially induced WSLs on extracted maxillary incisors that were photographed at different angles using standardized techniques. Their results showed non-significant interobserver differences in the surface measurements of WSLs of less than 0.1 mm [13].

#### Outcomes for implementation process evaluation

Professionals’ attitudes: The professionals’ attitudes toward using the CPG will be measured with a questionnaire. Each orthodontist using the CPG will be asked to respond on a five-point scale to questions relating to several factors that influence CPG adherence, as described by Grol et al. [15]. These items include influence on professional autonomy, legal aspects, quality of treatment, and implications for practice management. Readability and content validity of this questionnaire is being tested by an expert in the field of guideline adherence (MW) and two orthodontic academic staff members who work in private practice. After adjustments are made, the questionnaire will be used for the clinical study. Professionals utilizing the CPG will be asked to complete the questionnaire before starting the trial (after dissemination of the CPG and the clinical outreach visit), as well as at the end of the trial. Thus, we will also be able to evaluate any change in attitude after working with the CPG in routine practice.

Professionals’ compliance with CPG guidelines: The professionals’ compliance with following the CPG guidelines will be investigated by reviewing the EPF. In the Dutch health care system, all legal health care interventions are defined and priced, including the preventive measures included in the CPG, that is, oral health instruction and application of fluoride varnish. Therefore, registration of preventive measures in the EPF is automatically coupled to cost declarations. This will enable us to calculate the proportion of patients receiving the appropriate treatment/advice and the declared treatment costs in relation to the recorded individual caries risk.

Patients’ experience with preventive measures: The patients’ experience with the preventive measures will be investigated using a newly developed digital questionnaire that focuses on the following three aspects:

1. Did the patient receive the guideline-recommended instructions/preventive measures?
2. What is the patient’s opinion regarding the quality of the instructions and/or preventive measures?
3. Did the patient follow the instructions/preventive measures?

Prior to its dissemination in the trial, the questionnaire’s readability and content validity will be tested in a sample of ten orthodontically treated patients at the academic Department of Orthodontics of the Radboud University Medical Center. Patients will be asked to complete the questionnaire in the presence of the main investigator, enabling us to gain more insight regarding parts of the questionnaire that may be difficult, unclear, or incomplete. Adjustments will be made if needed.

### Data collection

The data collection and timeframe are given in Table 3.

**Table 3:** Data collection and study timeframe

Months	
1–2	14 orthodontic practices included
	Collection of baseline data (photographs of 50 treated patients and baseline questionnaire)
3	Assignment to intervention or control arm
	Information meeting
	Dissemination of CPG and educational outreach visit 1 week later*
	Professional questionnaire*
4–6	Start inclusion of patients with informed consent
10–12	Patient questionnaire
	EPF registrations
10–15	Intra-oral photographs (6–9 months into treatment)
22–30	Registration of compliance in electronic patient files*
	Intra-oral photographs
	Professional questionnaire*

* For intervention group only

#### Baseline data

After giving their informed consent to participate, orthodontists will be asked to complete a questionnaire including personal and practice information. They will also be asked about the usual preventive strategies applied in their practice. Additionally, we will evaluate pre- and post-treatment intra-oral pictures of the last 50 patients who completed orthodontic treatment with fixed appliances at each practice, to obtain the baseline incidence of WSLs at each participating orthodontic practice.

#### Intra-oral digital photographs

**Fig. 2:**
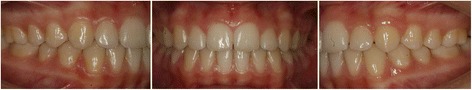
Intra-oral photographs

For all included patients, three intra-oral digital photographs will be taken (Fig. 2) at each of three time points: T0, pre-treatment before placing fixed appliances; T1, 6–9 months into treatment with fixed appliances; and T2, end of treatment with fixed appliances. Intro-oral photographs will be taken after polishing all tooth surfaces, and utilizing standardized lighting conditions and camera positioning. The timing for T1 was chosen because this is when a radiograph is usually taken to diagnose possible root resorption. Combining the radiographic procedure with taking intra-oral digital photographs reduces the interference with routine practice and the additional burden to the patient. In most orthodontic practices, it is standard procedure to take intra-oral photographs before (T0) and after treatment (T2); therefore, these photographs do not represent an additional burden to either the patient or the orthodontist. An orthodontic treatment usually lasts about 2 to 2.5 years.

#### Professionals’ attitude toward working with the guideline

To evaluate the attitudes of professionals working with the CPG, these orthodontists will be asked to complete a questionnaire. The main investigator will personally hand the questionnaire to these practitioners during the outreach visit, after providing all information regarding use of the CPG. The same procedure will be repeated at the end of the trial. If a practitioner fails to respond, a reminder will be sent after 2 weeks.

#### Patients’ experience with preventive strategies

Patients will be asked to complete a questionnaire asking their opinion about the preventive strategies used by their practice. The questionnaire will be sent to all included patients at 6 months after inclusion of the first patients. At that point, the first and last included patients will have undergone approximately 6 and 4 months of treatment, respectively. Two weeks after the questionnaire is mailed, a reminder will be sent.

#### Compliance with working with the guideline

Orthodontists in the intervention group will use the CDSS integrated in the EPF. At each check-up visit, the computer will ask the orthodontist to register the patient’s oral health status and oral hygiene status. Subsequently, the program will generate the preventive measures to be applied following the recommendations of the CPG. This information will then be automatically stored in the EPF. In the control arm of the study, only oral health and hygiene status at the start of treatment will be registered.

### Informed consent

Before starting treatment, all patients in both the intervention and control practices will receive a general letter containing information about the study. Patients will be informed that if they participate, they will be asked to complete a questionnaire, and information regarding oral health and oral hygiene will be extracted from their patient files. Patients at the intervention practices will be given specific information regarding the use of the CPG as part of an informed consent letter that will be signed by the patient and their parents prior to orthodontic treatment. Patients at the control practices will be receiving usual preventative care and will not be asked to give informed consent for the preventive measures used.

### Statistical analysis

#### Reliability of WSL recording

Kappa values will be calculated to evaluate the inter-rater agreement for recording WSLs from intra-oral photographs. For this purpose, ten pre- and post-treatment photographs per orthodontic practice will be analyzed independently by two experienced orthodontists.

#### Primary outcome

To account for the data clustering (in orthodontic practices), a multilevel logistic regression analysis will be applied on patient data, using the presence of new WSLs at T2 (yes/no) as the outcome, and the type of intervention (CPG or usual preventive measures) as the predictor. In this model, the regression coefficient of the predictor “intervention versus control” indicates the effectiveness of the intervention. Given the expected distribution of outcomes, we anticipate the use of binomial logistic regression with a logit link function. We will also examine the influences of age at start of treatment, gender, patients’ opinion on preventive measures, patients’ initial oral health status, patients’ initial oral hygiene status, and patients’ initial sugar intake on the primary outcome measure, by including these factors one by one as predictors in the regression model. These covariates are known to potentially influence the development of WSLs during orthodontic treatment [3, 9]. We do not anticipate a high number of missing values. However, in the event that we do have high numbers of missing values, we will apply a multiple imputation method prior to the planned analyses.

#### Secondary outcome: evaluation of implementation

The professionals’ attitudes toward working with the CPG will be evaluated using descriptive statistics. Additionally, McNemar tests will be used to compare the attitude at the start of the trial with that at the end of the trial. Since the study was not powered for this outcome measure, the results are only explorative. The professionals’ compliance with working with the CPG will also be evaluated using descriptive statistics. For the intervention group, we will additionally evaluate the influence of the professionals’ compliance on the incidence of WSLs. Chi-square tests will be used to compare the patients’ experiences with the preventive strategies used (CPG versus usual strategies).

## Discussion

This study will investigate the effectiveness of a newly developed guideline to improve oral health during orthodontic treatment, while simultaneously illuminating potential difficulties in adopting a guideline in general orthodontic practice. The innovative features of this study include the risk-based CDSS that discriminates between patients’ oral health statuses with regard to preventive measure utilization in general orthodontic practices. Most studies focusing on WSL prevention apply the preventive intervention to each patient in an experimental setting, resulting in overtreatment and a disconnect from the real-world conditions in which the intervention is to be applied [16, 17]. Additionally, one of the overreaching goals of this initiative is to create a gold standard for WSL prevention during orthodontic treatment, against which future studies can compare new promising preventive measures and the readiness of clinicians to change and adopt new treatments. By doing so, we want to help bridge the gap between science and orthodontic clinical practice and improve the quality of oral health care [18, 19].

### Trial status

Six practices have been recruited. Baseline data regarding WSL incidence and preventive strategies have been analyzed. At the present phase of the trial, we are actively recruiting the remaining eight practices.

### Consent

Written informed consent was obtained from the patient for the publication of this manuscript and the accompanying images. A copy of the written consent is available for review by the Editor-in-Chief of this journal.

## Abbreviations

CDSS: computerized clinical decision support system
CPG: clinical practice guideline
EPF: electronic patient file
WSL: white spot lesion
